# Controlling Syneresis of Hydrogels Using Organic Salts

**DOI:** 10.1002/anie.202115021

**Published:** 2021-12-07

**Authors:** Santanu Panja, Bart Dietrich, Dave J. Adams

**Affiliations:** ^1^ School of Chemistry University of Glasgow Glasgow G12 8QQ UK

**Keywords:** Co-assembly, Multicomponent hydrogel, Rheology, Syneresis

## Abstract

Supramolecular hydrogels can spontaneously undergo syneresis through fibre–fibre interactions and expel significant amounts of water upon aging. In this process, the hydrophobicity of fibres which regulates the 3D‐rearrangement of the self‐assembled structures during syneresis is important. Here, we show that we can control the hydrophobic microenvironment of gels by incorporating organic salts into the co‐assembled gel fibres thereby enabling control of the macroscopic gel volume phase transition.

## Graphical abstract

For self‐shrinkable hydrogels, syneresis can be controlled by tuning the hydrophobic microenvironment within the gel network involving co‐assembly with organic salts.

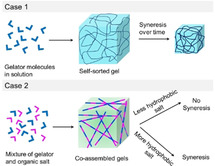

Supramolecular hydrogels derived from the self‐assembly of small organic molecules (called gelators) in water have applications in areas including drug delivery, tissue engineering, catalysis, and optoelectronics.^[1]^ Typically, self‐assembly of gelators results in fibres which are maintained by different non‐covalent interactions such as hydrogen bonding, stacking, ionic and hydrophobic interactions.^[2]^ Such non‐covalent interactions are weak and reversible which makes supramolecular gels highly responsive to stimuli like heat, pH, ions, UV‐light, and electrical and magnetic fields, causing a change in optical as well as mechanical properties of the materials.^[3]^

Syneresis is the process of contraction in gel volume accompanied by expulsion of the solvent that was initially entrapped by the self‐assembled network.^[4]^ Syneresis is observed in living systems, for example, release of blood serum during the clotting process.^[5]^ Syneresis is also common in the food industry, for example, in jams, jellies, tomato juice and dairy products.^[6]^ In synthetic supramolecular hydrogels, syneresis is mostly driven by the change in hydrophobicity of the gelators in response to an applied (or external) stimulus.^[7]^ Enhanced hydrophobicity of the components results in increase in hydrophobicity of the self‐assembled fibres followed by expulsion of water from the gel network. Since the first report of thermally induced shrinking of supramolecular gels by the Hamachi group in 2002,^[8]^ stimulus‐triggered supramolecular gels with a macroscopic volume phase transition property have been receiving interest for their potential in devising actuators, smart switchable materials, artificial muscles, drug release, biosensing, microfluidic devices, and water purification.[^7^, ^9^] Such gels are stable under normal conditions and only undergo syneresis when exposed to specific stimuli. Consequently, the mechanical properties like rigidity, gel strength, and self‐healing behaviour of the syneresed gels differ significantly from the original materials.

In contrast, there are only a few gels that show shrinking behaviour without an external stimulus.^[10]^ In such systems, a metastable gel is initially formed which spontaneously undergoes syneresis upon aging. The Banerjee group reported a tripeptide‐based gel and linked the syneresis to the inherent hydrophobicity of the gelator.^[10b]^ However, this is not always true. For a series of Fmoc‐dipeptides, while hydrophobic dipeptides form stable, self‐supporting materials, gels formed by less hydrophobic compounds tend to exhibit syneresis.^[11]^ Hence, in this case, syneresis is strongly related to the hydrophobic microenvironment of the gels, as opposed to simply the hydrophobicity of the molecules.^[12]^ There is possibly an evolution of the underlying network structures to a more densely packed network through fibre–fibre interactions.[^12^, ^13^] The change in the 3D‐arrangement of the self‐assembled structures driven by the hydrophobicity of the network causes macroscopic contraction of gels.[^10c^, ^13^]

The hydrophobic microenvironments of gels have been found to be crucial in stabilizing encapsulated enzymes, proteins etc.^[14]^ We hypothesized that controlling the hydrophobic microenvironment within the gel network by chemical additives might enable controlling of the syneresis. To verify our proposition, here we describe the effect of addition of organic salts on the syneresis of a dipeptide hydrogel. Previously, Qin et al. demonstrated that addition of metal ions to an amphiphilic dendron gel can trigger volume phase transition at a macro‐level by changing the hydrophobicity of the self‐assembled fibres.^[7d]^ A similar observation by the Liu group described imposing syneresis on a stable gel by the addition of Cu^2+^ ions.^[7e]^ However, our present study has a different (and opposite) goal to these reported systems; we prevent syneresis of supramolecular gels by using structurally similar yet different hydrophobic organic salts (Figure 1), and control the final properties of the materials. It is noted that for the first time this has been done for supramolecular low‐molecular weight gels, but there are precedents with polymer systems.^[15]^

**Figure 1:**
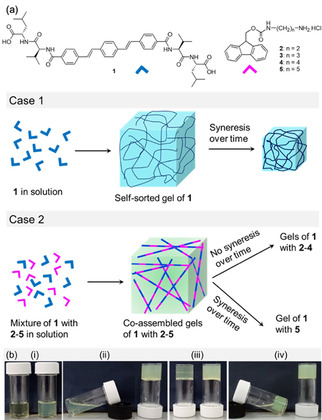
a) Cartoon showing the network formation by **1** in the absence and presence of organic salts **2**–**5**. The extent of syneresis of the resulting co‐assembled gels depends on the hydrophobicity of the Fmoc‐salts. b) Photographs showing the phase changes of i) the solutions of **1** and (**1**+**2**) in presence of GdL after ii) 1 h, iii) 3 h and iv) 18 h. In each photograph, the left and right vials represent the systems **1** and (**1**+**2**), respectively. Concentrations of **1** and **2** are 5 mg mL^−1^ and 2 mg mL^−1^, respectively. Initial concentration of GdL is 5 mg mL^−1^.

The previously reported gelator (**1**, Figure 1) forms gels at low pH that undergo syneresis.^[13]^ Initially, a solution of **1** was prepared by dissolving the compound in water in the presence of NaOH (2 molar equivalents). The pH of the solution was then adjusted to pH 10. At this pH, compound **1** produced a free flowing solution.^[13]^ To trigger the gelation, the pH is decreased using base‐catalysed hydrolysis of glucono‐d‐lactone (GdL) to gluconic acid.^[16]^ Addition of GdL (5 mg mL^−1^) to the alkaline solution of **1** (5 mg mL^−1^) at pH 10 resulted in a gradual reduction of the pH sufficiently below the apparent p*K*_a_ of **1** (final pH is 3.7) (Figure 2, Figure S1). A self‐supporting gel was formed initially, which underwent syneresis on aging (Figure 1b).^[13]^

**Figure 2:**
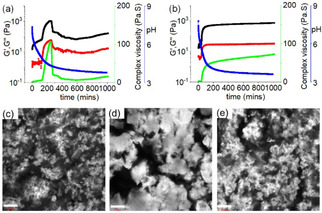
a, b) Variation of pH (blue), G′ (black), G′′ (red) and complex viscosity (green) with time for **1** (a) and the mixture of **1** and **2** in the presence of GdL in water (b). c, d) Confocal fluorescence microscopy images of the hydrogel of **1** after 4 h (a) and 18 h (b). e) Confocal fluorescence microscopy images of the hydrogel of (**1**+**2**) after 18 h. In all cases, the scale bar is 20 μm. In all cases, concentrations of **1** and **2** are 5 mg mL^−1^ and 2 mg mL^−1^, respectively. Initial concentration of GdL is 5 mg mL^−1^.

We probed the development of the gel using rheology (Figure 2a). Correlation of the pH–time profile to that of the time sweep revealed that gelation begins after ≈1.5 hours at a pH below the p*K*_a_ of **1** which was evident from the increase of rheological moduli (G′ and G′′) and complex viscosity values. The 3D network became stronger as the pH decreased, and an invertible gel was obtained after ≈3 hours. During the process, the rheological moduli reached their maximum values after ≈4 hours. At longer times, a decrease in G′ and G′′ was recorded until they reached a plateau after ≈15 hours. The variation in complex viscosity followed a similar trend to that of rheology. The decrease in rheological moduli after formation of the gel (after ≈4 hours) can be ascribed to the gel undergoing syneresis.^[13]^ The gel expelled ≈58 % of the entrapped water after ≈18 hours upon syneresis (Figure 1b). Under confocal microscope, the non‐syneresed gel of **1** (after ≈4 hours) showed closely spaced small spherulitic domains of fibres (Figure 2c). In the shrunken gel (after ≈18 hours), the density of spherulitic structures decreased and large aggregates were formed (Figure 2d). Syneresis of **1** is due to an evolution of the underlying fibres to a more densely packed network through fibre‐fibre interactions.^[13]^ The gel shrank three‐dimensionally and adopted the shape of the container (Figure 1b, Figure S1).

With the aim to influence hydrophobicity of the self‐assembled fibres of **1** involving co‐assembly, a series of hydrochloride salts of Fmoc‐monoprotected alkyldiamines (**2**–**5**) were used (Figure 1). We hypothesized that intermolecular hydrogen bonding between **1** and **2**–**5** involving the amide functionalities can promote the co‐assembly. Furthermore, aromatic stacking between the oligophenylene vinylene core of **1** and the Fmoc‐groups of **2**–**5** can also contribute to the co‐assembly formation. However, hydrophobicity of the co‐assembled fibres is expected to be different with increase in hydrophobic structural features of the salts from **2** to **5**.^[17]^ Initially, we used compound **2** to perturb the gelation of **1** (Figure 1). The ammonium salt **2** is highly soluble in water exhibiting a pH of around 5.2 (Figure S2). Compounds **1** and **2** show opposite responses on pH perturbation. At a pH above the p*K*_a_ of **2** (p*K*_a_ is 8.4),^[18]^ formation of corresponding amine results in gelation (Figure S2).^[18]^ Compound **2** was stable at high pH and did not undergo deprotection of the Fmoc‐group which was confirmed by ^1^H NMR with no peak near 6.2 ppm corresponding to the olefinic protons of bibenzofulvene (Figure S3).^[18]^ When an aqueous solution of **2** (2 mg mL^−1^, 1 molar equivalent with respect to **1**) was added to **1** (at pH 10), the resulting mixture exhibited a pH of ≈9.3. Although the pH of the mixture of (**1**+**2**) was higher than the apparent p*K*_a_ of **2**, no gel formation was observed suggesting sufficient interaction between the molecules of **1** and **2** that gelation at high pH was inhibited (Figure 1b). To confirm this, fluorescence and UV/Vis studies were conducted with **1** and **2** at high pH. By fluorescence, while compound **2** showed a broad emission band near 390 nm at high pH, an alkaline solution of **1** exhibited strong emission at 480 nm (Figure S4, S5). In presence of **2**, the emission of **1** was blue shifted by 8 nm (Figure S5). Similarly, in the UV/Vis spectrum of the mixture of (**1**+**2**), the absorption signature of **1** at 347 nm was blue shifted by 10 nm and appeared at 337 nm (Figure S5). These results corroborate substantial interaction between **1** and **2** at high pH. Moreover, in the ^1^H NMR of the mixture of (**1**+**2**), significant broadening of the aromatic protons of **1** and **2** indicates co‐assembly formation at high pH (Figure S6).

When GdL was used to trigger gelation of **1** in presence of **2**, a gel was formed as the pH further decreased over time (Figure 1b). There was an increase in the rate of pH decrease for the mixture of (**1**+**2**), although the final pH for the binary system was identical to that of the system of **1** alone (Figure S7). Substantial changes were noticed in time sweep rheology and in visual appearances of the gels (Figure 1b, Figure 2 b). The gel appeared quickly in presence of **2** (after ≈1 hour). This was verified by time variable rheology experiments which showed that the increase of rheological moduli and complex viscosity for the (**1**+**2**) system was faster than the system of **1** alone. The rheological moduli started to increase after 40 minutes for the mixture of (**1**+**2**) and reached stable values after ≈4 hours. Interestingly, in presence of **2**, the hydrogel of **1** did not show any sign of syneresis over the course of the experiments; there was no release of water from the gel even after 5 days (Figure 1b, Figure S7). Time sweep rheology also backs the visual observation as there were no further changes of G′ and G′′ with time after reaching the stable values (Figure 2b).

The amine of **2** undergoes protonation with the pH decrease producing the less hydrophobic hydrochloride salt. It has been reported that presence of salts can affect the hydration of peptides involving the Hofmeister effect.^[19]^ To investigate this, spectroscopic studies were conducted with the syneresed gel of **1** and the hydrogel of (**1**+**2**) (Figure 3). The shrunken gel of **1** exhibited emission at 468 nm, a 12 nm blue shifted band compared to the emission of **1** at high pH. In contrast, reduction of pH resulted in 2 nm red shifts (from 472 nm to 474 nm) in emission for the mixture of (**1**+**2**). Correlation of emission profiles of the gels at low pH reveals that, in the presence of **2**, the emission of **1** was red shifted by 6 nm. Similarly, by UV/Vis, the absorption of the syneresed gel of **1** at 345 nm was red shifted to 347 nm along with increase in intensity in the region 404 nm for the hydrogel of (**1**+**2**). Additionally, the shoulder peak at 245 nm in the shrunken gel of **1** merged with the absorption of **2** at 265 nm and appeared at 263 nm in the hydrogel of (**1**+**2**) (Figure 3a, S4). These results suggest that significant interactions were present between **1** and **2** at low pH. To infer the intermolecular interactions between **1** and **2** in the gel, FTIR studies were conducted (Figure S8). In the FTIR of the syneresed gel of **1**, amide carbonyl stretching appeared in the range 1648–1623 cm^−1^. Different intensities of the peaks in the region 1648–1623 cm^−1^ imply co‐existence of varieties of H‐bonding stack sizes.^[20]^ In the presence of **2**, while the peak at 1648 cm^−1^ remained unaffected, the shoulder at 1623 cm^−1^ shifted to 1620 cm^−1^ with enhanced intensity. Although subtle, these changes in the amide carbonyl stretching of **1** in presence of **2** suggest hydrogen bonding interactions between **1** and **2** in the gel. Due to intermolecular hydrogen bonding between **1** and **2**, the amide NH stretches of **1** near 3386 cm^−1^ moved to a lower region by 33 cm^−1^ in presence of **2**. Hence, gelator **1** undergoes co‐assembly with **2** and thereby influences the gelation kinetics and also the material properties (Figure 1a). The co‐assembly between **1** and **2** in the gel was also confirmed by time variable ^1^H NMR experiments (Figure S6). Upon addition of GdL to the mixture of (**1**+**2**) at high pH, gradual disappearance of the signals for the aromatic protons of both **1** and **2** suggests a sol to gel transition with the pH decrease. After 16 h, the aliphatic protons of both **1** and **2** became too broad to distinguish. These results emphasize co‐assembly formation between **1** and **2** in the gel where almost 100 % of both the components were involved in fibre formation. There was a difference in the microstructure of the gels. The hydrogel of (**1**+**2**) showed a higher density of spherulitic structures than the syneresed gel of **1** (Figure 2c–e). The microstructure of the gel of (**1**+**2**) was very similar to the microstructure of the non‐syneresed gel of **1** (after ≈4 hours). We hypothesize that accumulation of **2** into the gel network results in a decrease in hydrophobicity of the fibres. Such a change in the hydrophobic environment of the gel matrix prevents syneresis.

**Figure 3:**
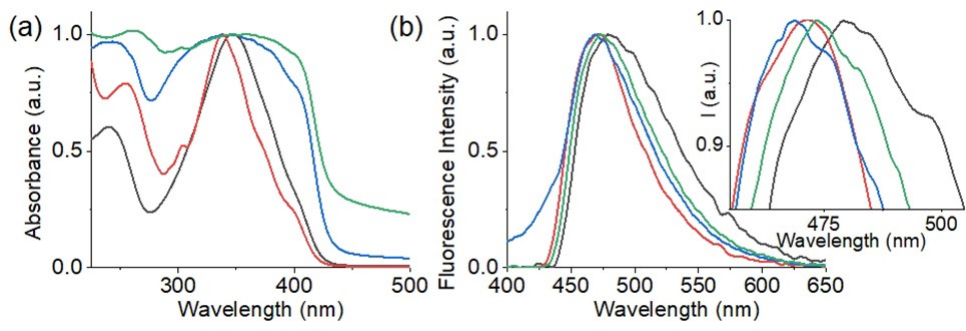
a) Normalized absorption and b) normalized emission spectra (*λ*_ex_ is 350 nm) of **1** and **2** under different conditions. In both cases, the black and red data represent the solutions of **1** and (**1**+**2**), respectively; the blue and green data represent the syneresed gel of **1** and the hydrogel of (**1**+**2**), respectively. Inset of figure (b) represents an expanded region of graph (b). In all cases, concentrations of **1** and **2** are 5 mg mL^−1^ and 2 mg mL^−1^, respectively. Initial concentration of GdL is 5 mg mL^−1^.

To verify our proposition, we gradually increased the chain length of the ammonium salt (compounds **3**–**5**) and investigated the effect of increasing hydrophobicity on the resulting co‐assembled gels (Figure 1). Compounds **3**–**5** have similar pH‐responsive properties to those of **2** in solution. However, unlike **2**, none of the amine forms of **3**–**5** formed a gel under basic conditions and instead precipitated (Figure S2). In the presence of **1**, all the compounds **3**–**5** produced solutions at high pH (Figure S9). The absorption and emission spectra for the mixtures of **1** with (**3**–**5**) were almost identical to that of (**1**+**2**) (Figure S10). On addition of GdL to the individual mixtures of **1** with (**3**–**5**), again the pH gradually reduced to ≈pH 3.7 with no substantial difference in the pH–time profile compared to the (**1**+**2**) system (Figure 2b, S11). However, by time sweep rheology, the behaviour of the mixture of (**1**+**5**) was different to the rest of the binary systems. Rheological studies showed that there was little change in G′ and G′′ after reaching stable values (after ≈4 hours) for the mixtures of (**1**+**3**) and (**1**+**4**), as for the (**1**+**2**) system. In contrast, for (**1**+**5**), the rheological moduli and complex viscosity values started to decrease after ≈4 hours until they reached a plateau after ≈14 hours. Visually a stable gel was obtained from the mixtures of **1** with **3** and **4** with no syneresis, whereas the mixed gel of (**1+5**) expelled ≈29 % of solvent from the system after ≈18 hours (Figure S12). While the microstructures of the gels of (**1**+**3**) and (**1**+**4**) were like that of (**1**+**2**), the shrunken gel of (**1**+**5**) exhibited spherulitic aggregates like **1** alone (Figure S13). This indicates that syneresis in the gel (**1**+**5**) was due to the increase in hydrophobic structural features of the salts. Accumulation of **5** into the network increases the hydrophobicity of the co‐assembled fibres and influences the macroscopic behaviour of the gels. On increasing hydrophobic chain length of amines, the sharp emission of the hydrogel of (**1**+**2**) at 474 nm gradually became broad and shifted to 478 nm in the hydrogel of (**1**+**5**) (Figure S14). However, the UV/Vis, and FTIR spectra of all binary systems (**1** with **2**–**5** at low pH) were similar (Figure S14, S15).

Oscillatory strain and frequency sweeps were conducted (≈18 hours after addition of GdL) to evaluate the mechanical properties of the different gels (Figure S16). All the resulting binary gels exhibited higher strain bearing capacity than the gel of **1** (critical strain or gel strength, the strain at which the gel breaks, increases from ≈3 % to 6–10 % in presence of salts). The stiffness (G′) of the gel of **1** increased twofold in presence of **2**. On increasing hydrophobicity of the salts, the storage modulus of the gels gradually decreased up to the system (**1**+**4**) (Figure S17). On further increasing the hydrophobic chain length of the ammonium component (**1+5**), the stiffness of the resulting gel increased remarkably. In frequency sweep, while the syneresed gel of **1** and the hydrogels of (**1**+**2**) and (**1**+**3**) exhibited frequency thinning in the high frequency region, the rheological moduli for the binary gels (**1**+**4**) and (**1**+**5**) were independent of frequency (Figure S16). These results imply that not only the volumetric properties but also the mechanical behaviour of the gels can be tuned by varying the hydrophobicity of the salts.

It is further possible to control self‐shrinking of **1** by varying the proportions of the components. As an example, we gradually reduced the concentration of **2**. When the concentration of **2** was reduced to 1 mg mL^−1^ (0.5 molar equivalent with respect to **1**), no syneresis was noticed (Figure S18). This gel exhibited similar microstructure to that of the gel obtained from the mixture of **1** with high concentration of **2** (Figure S19). However, on further reduction of concentration of **2** to 0.5 mg mL^−1^ (0.25 molar equivalent with respect to **1**), again syneresis occurred with the expulsion of ≈42 % of water and the syneresed gel exhibited similar microstructure to the shrunken gel of **1** (Figure S18, 19). We conducted UV/Vis experiments with the expelled water obtained from different systems. From Figure S20, it is evident that after syneresis of gels, characteristic absorbance of **1** at 350 nm (Figure 3c) was absent in the expelled solutions indicating complete accumulation of **1** in the shrunken gels. However, the exuded solutions from the binary gels of (**1**+**5**) and (**1**+**2**) exhibited very weak absorption at 265 nm characteristic of the respective ammonium salts. By analysing the absorption spectra, it was found that only 0.33 % and 2 % of the salts **5** and **2** were released after syneresis, respectively. As an application, our method can be utilized to prepare a monolith of gel where one side only contains the organic salt. This approach can be used to prepare a system where one hemisphere of gel undergoes syneresis while the other hemisphere of gel containing the Fmoc‐salt retains its initial volume. Using two pH‐sensitive dyes allows the change in the volume of the gels to be visualized. This is schematically demonstrated in Figure 4.

**Figure 4:**
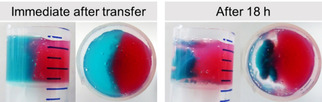
Time variable changes in two hemispheres prepared from gels formed from **1** in the absence (left) and presence (right) of **2**. In both cases, concentrations of **1** and GdL are 5 mg mL^−1^. For right hemisphere, concentration of **2** is 2 mg mL^−1^. Nile blue (left) and methyl red (right) were used to dye the gels ([dye] is 0.25 mg mL^−1^).

We also investigated the effect of other amines on the syneresis of **1**. For these experiments, we used different aliphatic and aromatic ammonium salts (Figure 5a), and the results are described in Figure 5b and Figure S21–23. None of the salts have gelling ability either alone or in presence of base (Figure S21). From Figure 5b, it can be concluded that the amounts of expelled water and so the final volume of the hydrogel of **1** can be controlled by incorporating both aliphatic and aromatic ammonium salts. By time sweep rheology, while the variations of G′, G′′ and complex viscosity for the system (**1**+**7**) exhibited similar trends as that of **1** alone (Figure S21, Figure S23), no decrease in rheological moduli and complex viscosity was observed for (**1**+**9**) after reaching their stable values (similar to (**1**+**2**)). These results imply that ammonium salts with large π‐surface are more effective at preventing the syneresis. Finally, to verify if our method is applicable to other hydrogel systems, we carried out similar experiments with gelator **10** (Figure 5c). Like **1**, the hydrogel of **10** also undergoes syneresis expelling ≈5 % of water after 18 h. In presence of **2**, no syneresis was noticed. Again, time sweep rheology for the system (**10**+**2**) showed significant differences than the system of **10** alone with no deviations of rheological moduli and complex viscosity after 3 h (Figure S24). These results suggest that our methodology can be applied in general to prevent syneresis of hydrogels.

**Figure 5:**
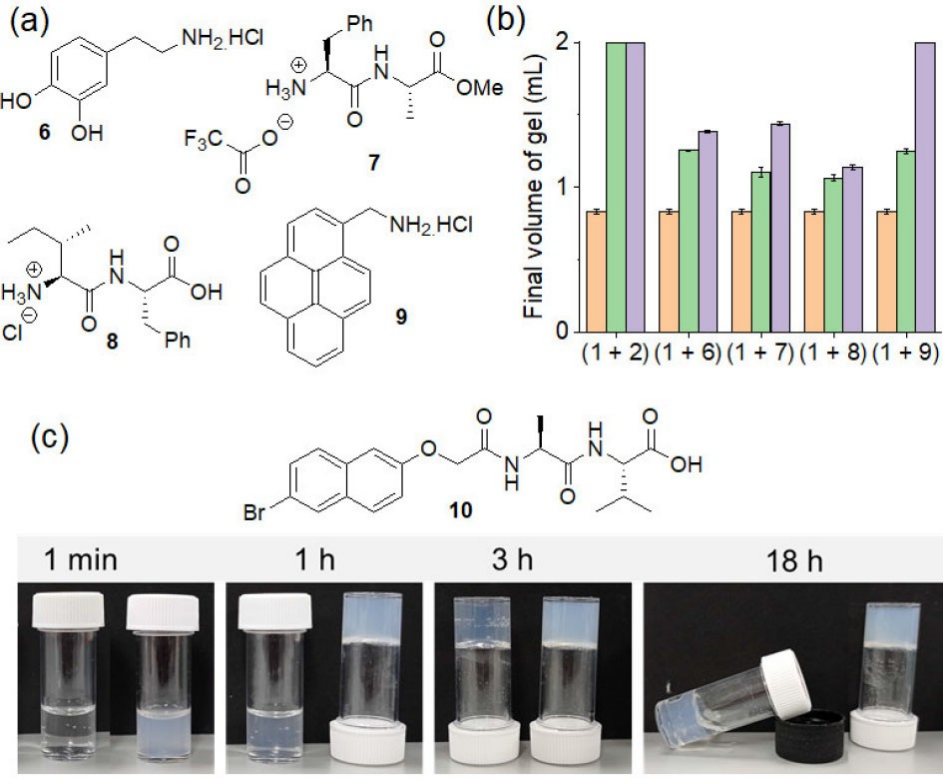
a) Chemical structures of the salts **6**–**9**. b) Bar graph representing final volume of the gels of **1** prepared in the absence (ochre) and presence of 0.5 molar equivalent (green) and 1 molar equivalent (purple) of different organic salts. In all cases, concentrations of **1** and GdL are 5 mg mL^−1^. c) Photographs showing the phase changes of the solutions of **10** and (**10**+**2**) in the presence of GdL with time. In each photograph, the left and right vials represent the systems **10** and (**10**+**2**), respectively. Concentration of **10** is 5 mg mL^−1^, concentration of **2** is 0.5 molar equivalent. Initial concentration of GdL is 5 mg mL^−1^.

In conclusion, we have demonstrated that the hydrophobic microenvironment within the gel network is crucial for a gel to undergo syneresis spontaneously. The hydrophobic microenvironment of a gel can be controlled by incorporation of different hydrophilic organic salts into the co‐assembled fibres. Furthermore, by varying the hydrophobicity or the concentration of the salt, it is possible to control the hydrophobicity of the resulting fibres and thereby syneresis of the gels. We have also shown that this methodology can be applied in general to prevent syneresis of hydrogels.

## Experimental Section

See Supporting Information for full experimental details, rheology, pH, UV/Vis, fluorescence, confocal microscopy, ^1^H NMR and photographs of gels.

## Conflict of interest

The authors declare no conflict of interest.

## Supporting information

As a service to our authors and readers, this journal provides supporting information supplied by the authors. Such materials are peer reviewed and may be re‐organized for online delivery, but are not copy‐edited or typeset. Technical support issues arising from supporting information (other than missing files) should be addressed to the authors.

Supporting Information

## Data Availability

The data that support the findings of this study are available from the corresponding author upon reasonable request.
