# Supplementary material for: Controlling Syneresis of Hydrogels Using Organic Salts
Source: Angew Chem Int Ed Engl. 2021 Dec 7;61(4):e202115021. doi: 10.1002/anie.202115021 (PMC9299832; doi:10.1002/anie.202115021)
Supplement: Supplementary file 1 — Supporting Information [file ANIE-61-0-s001.pdf]

Angewandte Chemie
Eine Zeitschrift der Gesellschaft Deutscher Chemiker

Supporting Information

Controlling Syneresis of Hydrogels Using Organic Salts

*S. Panja, B. Dietrich, D. J. Adams**

Table of Contents

Experimental Details	S3
Supplementary Figures	S11
References	S19

Experimental details

Materials: Compound **1**[1] and **10**[2] were synthesised as described previously. Compounds **2**-**6** and **9** were purchased from commercially available suppliers and used as received. Compounds **7** and **8** were synthesized according to the procedure described below. All other chemicals and solvents used were purchased from commercial suppliers and used as received. Deionised water was used throughout all experiments.

Methyl (2*S*) - 2 - [(2*S*) - 2 - {[(*tert* - butoxy)carbonyl]amino} - 3 - phenylpropanamido]propanoate (**HK-010**)

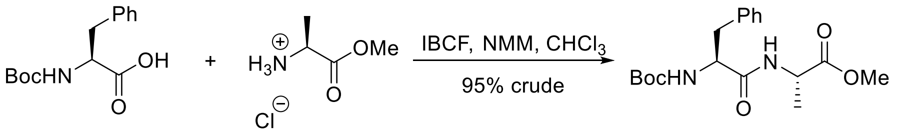

HK-010

To a solution of Boc-(*S*)-phenylalanine (8.40 g, 31.7 mmol) in chloroform (100 mL) was added *iso*-butyl chloroformate (1 eq, 4.11 mL) followed by *N*-methylmorpholine (1.1 eq, 3.83 mL). After stirring for 10 minutes, (*S*)-alanine methyl ester hydrochloride (1.02 eq, 4.51 g) and another portion of *N*-methylmorpholine (1.1 eq, 3.83 mL) were added and the reaction was left stirring overnight. After this time, it was diluted with chloroform, washed in turn with 1M hydrochloric acid, water, and brine, dried ($MgSO_4$), and evaporated to dryness under reduced pressure. Crude **HK-010** was thus obtained as an off-white solid (10.6 g, 95%) and used as is in the next step. A small amount was purified *via* column chromatography (1:9 ethyl acetate/dichloromethane) to afford an analytical sample. NMR data indicate a *ca.* 2:8 distribution of rotamers.

δ_H (400 MHz, DMSO-d_6) 8.44-8.40 (0.2H, m, NH), 8.37 (0.8H, d, *J* 6.98, NH), 7.33-7.24 (4H, m, H_{Ar}), 7.23-7.15 (1H, m, H_{Ar}), 6.87 (0.8H, d, *J* 8.67, NH), 6.44 (0.2H, d, *J* 9.04, NH), 4.35 (1H, m, CH^*), 4.22-4.14 (0.8H, m, CH^*), 4.12-4.05 (0.2H, m, CH^*), 3.62 (3H, s, OCH_3), 2.97 (1H, dd, *J* 13.80, 3.79, $PhCH_2$), 2.70 (1H, dd, *J* 13.91, 10.79, $PhCH_2$), 1.30 (3H, d, *J* 7.32, CH^*CH_3), 1.28 (7.5H, s, $C(CH_3)_3$), 1.22 (1.5H, s, $C(CH_3)_3$). δ_C (100 MHz, DMSO-d_6) 173.01, 171.77, and 155.24 (C=O), 138.19, 129.22, 127.98, and 126.15 (C_{Ar}), 77.95 ($C(CH_3)_3$), 55.38 (CH^*), 51.87 (OCH_3), 47.57 (CH^*), 37.37 ($PhCH_2$), 28.13 ($C(CH_3)_3$), 16.95 (CH^*CH_3). HRMS (ESI) m/z: $[M+Na]^+$ calcd for $C_{18}H_{26}N_2NaO_5$ 373.1734; found 373.1735.

^{1}H NMR of HK-010

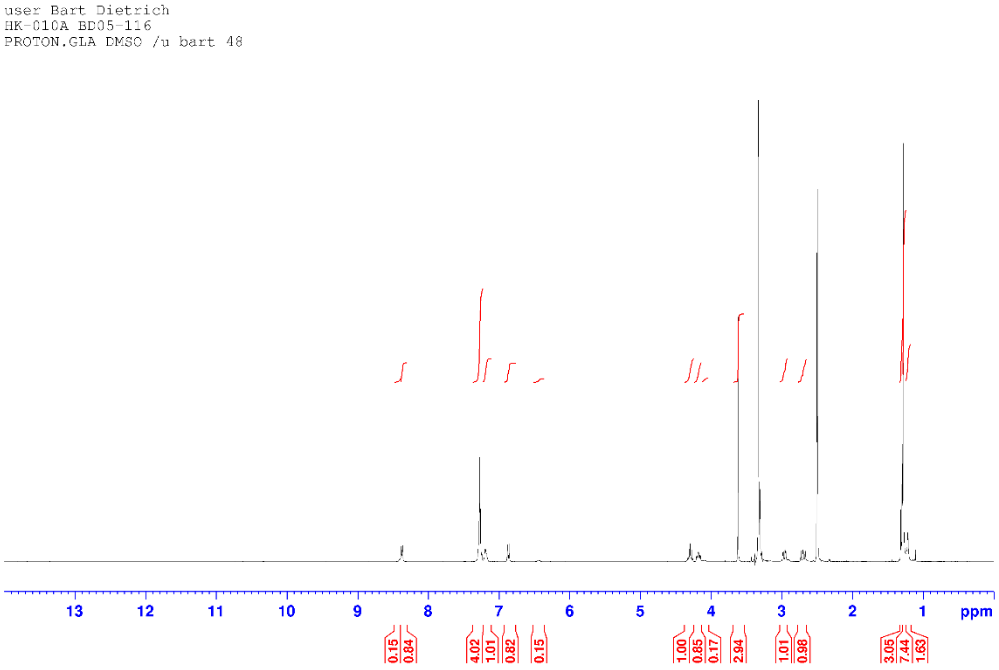

^{13}C NMR of HK-010

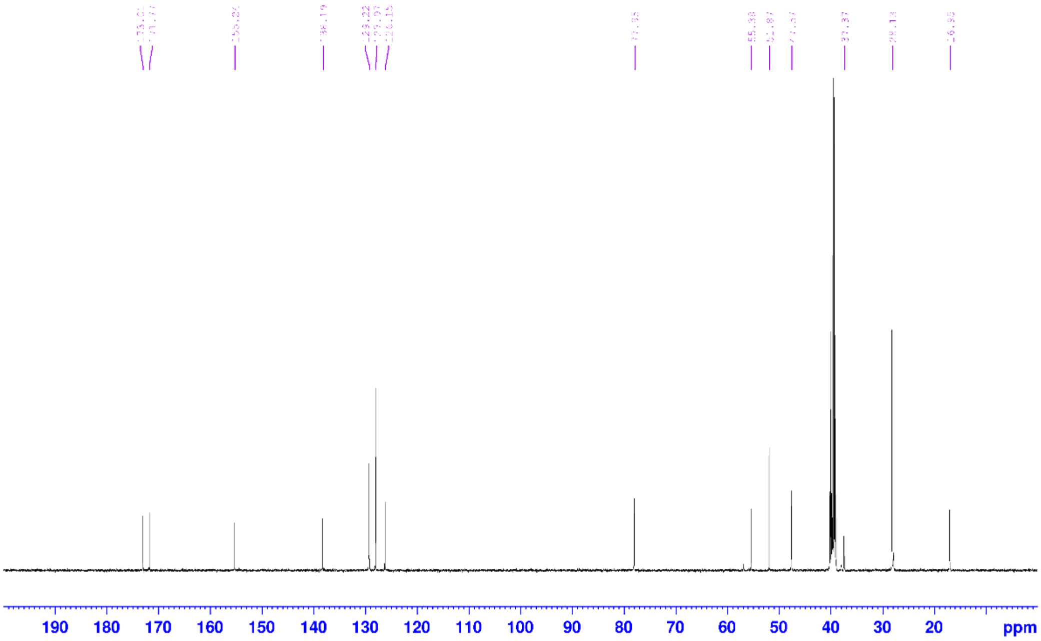

Methyl (2*S*) - 2 - [(2*S*) - 2 - amino - 3 - phenylpropanamido]propanoate trifluoroacetate (**7**)

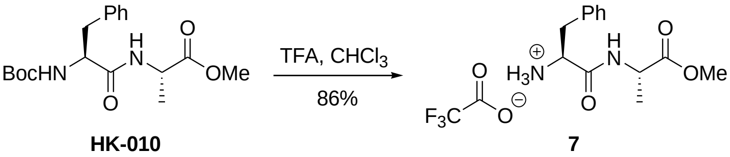

To a solution of **HK-010** (10.5 g, 30.0 mmol) in chloroform (40 mL) was added trifluoroacetic acid (25 mL, *ca.* 11 eq) and the mixture was stirred overnight. It was then poured into excess diethyl ether and stirred for an hour. The precipitate was filtered off, washed in the filter with some diethyl ether, transferred in a conical flask and stirred vigorously with diethyl ether for a further hour. Another filtration, washing with diethyl ether, and drying under vacuum, afforded **7** as a white solid (9.41 g, 86%). NMR shows a small amount of residual diethyl ether.

δ_H (400 MHz, DMSO-d$_6$) 8.93 (1H, d, *J* 7.11, N<u>H</u>), 8.19 (3H, br s, N<u>H</u>$_3^+$), 7.38-7.21 (5H, m, <u>H</u>$_{Ar}$), 4.41 (1H, m, C<u>H</u>*), 4.06-3.98 (1H, m, C<u>H</u>*), 3.63 (3H, s, OC<u>H</u>$_3$), 3.12 (1H, dd, *J* 14.08, 5.76, PhC<u>H</u>$_2$), 2.94 (1H, dd, *J* 14.07, 7.77, PhC<u>H</u>$_2$), 1.31 (3H, d, *J* 7.27, CH*C<u>H</u>$_3$). δ_C (100 MHz, DMSO-d$_6$) 172.35, 167.96, and 158.38 (q, *J* 31.25) (<u>C</u>=O), 134.89, 129.62, 128.52, and 127.16 (<u>C</u>$_{Ar}$), 117.25 (<u>C</u>F$_3$), 53.29 (<u>C</u>H*), 52.08 (O<u>C</u>H$_3$), 47.76 (<u>C</u>H*), 36.86 (Ph<u>C</u>H$_2$), 16.92 (CH*<u>C</u>H$_3$). HRMS (ESI) m/z: [M-TFA+Na]$^+$ calcd for $C_{13}H_{18}N_2NaO_3$ 273.1210; found 273.1212.

^{1}H NMR of **7**

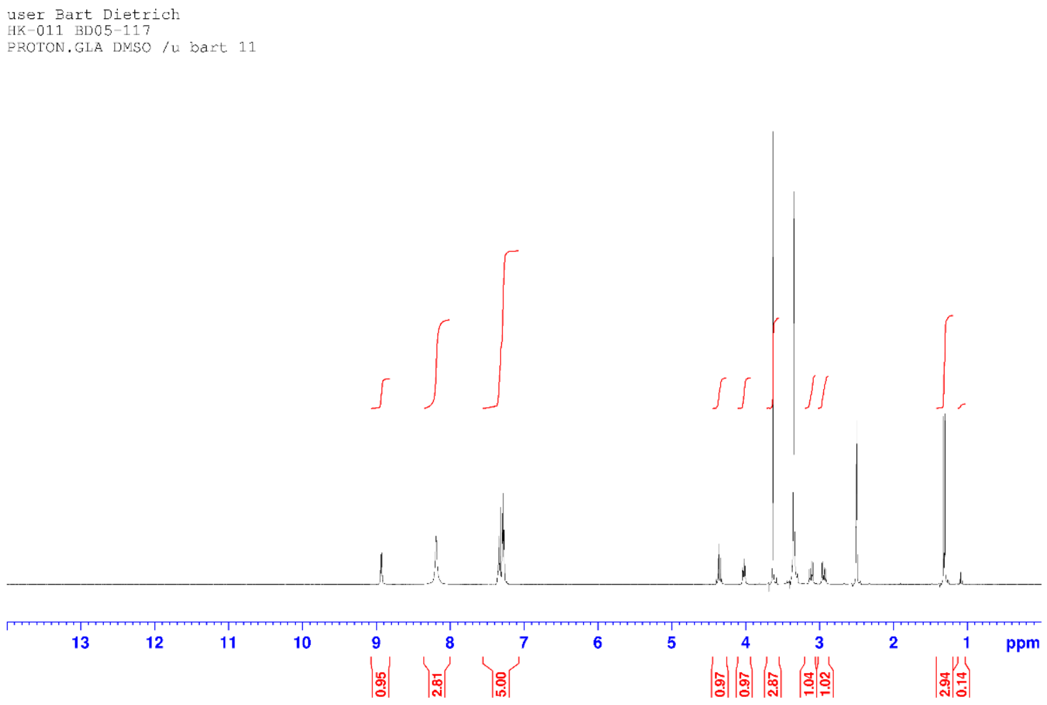

^{13}C NMR of **7**

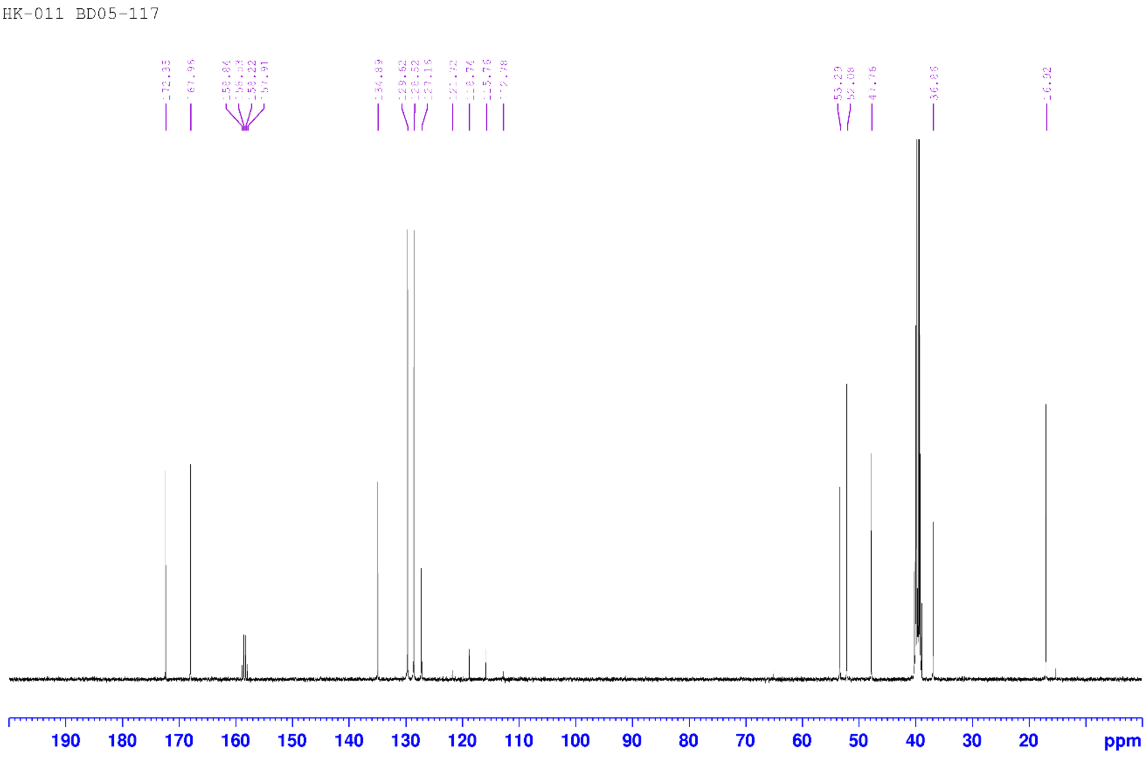

tert-Butyl (2S)-2-[(2S,3S)-2-{[(tert-butoxy)carbonyl]amino}-3-methylpentanamido]-3-phenylpropanoate (**FM-002**)

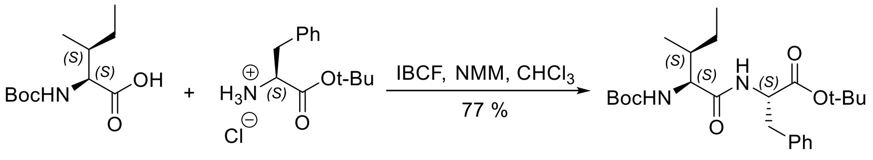

FM-002

To a solution of Boc-*iso*leucine (4.54 g, 19.6 mmol) in chloroform (50 mL) was added *N*-methylmorpholine (2.2 eq, 4.74 mL) followed by *iso*butyl chloroformate (1 eq, 2.68 g). After 10 minutes, phenylalanine *tert*-butyl ester hydrochloride (1.05 eq, 5.31 g) was added and the reaction was stirred at ambient temperature overnight. It was then diluted with chloroform, washed in turn with 1M hydrochloric acid, water, and brine, dried ($MgSO_4$), and evaporated under reduced pressure. Recrystallisation of the residue from boiling acetonitrile afforded the title compound as a white solid (6.56 g, 77%). Residual acetonitrile is seen in both proton and carbon NMR spectra.

δ_H (400 MHz, DMSO-d$_6$) 8.17 (1H, d, *J* 7.58, NH), 7.27-7.18 (5H, m, H_{Ar}), 6.64 (0.9H, d, *J* 9.26, NH), 6.25 (0.1H, d, *J* 9.24, NH), 4.37 (1H, dd, *J* 14.79, 7.59, O=C-CH*), 3.83 (0.9H, dd, *J* 8.92, 7.95, O=C-CH*), 3.71 (0.1H, m, O=C-CH*), 2.96 (1H, dd, *J* 13.89, 6.75, PhCH_2), 2.90 (1H, dd, *J* 13.88, 8.37, PhCH_2), 1.66-1.55 (1H, m, H_3C-CH*), 1.37 (9H, s, C(CH_3)$_3$), 1.34-1.28 (1H, m, CH_3-CH_2), 1.30 (9H, s, C(CH_3)$_3$), 1.10-0.96 (1H, m, CH_3-CH_2), 0.77 (3H, t, *J* 7.42, CH_3-CH_2), 0.74 (3H, d, *J* 6.78, H_3C-CH*). δ_C (100 MHz, DMSO-d$_6$) 171.26, 170.42, and 155.18 (C=O), 137.14, 129.18, 128.09, and 126.42 (C_{Ar}), 80.55 and 77.93 (C(CH$_3$)$_3$), 58.52 and 53.92 (O=C-CH*), 36.97 (PhCH_2), 36.69 (H_3C-CH*), 28.17 and 27.47 (C(CH_3)$_3$), 24.26 (CH_3-CH_2), 15.20 (CH_3-CH*), 10.92 (CH_3-CH_2). HRMS (ESI) m/z: [M+Na]$^+$ calcd for $C_{24}H_{38}N_2NaO_5$ 457.2673; found 457.2663.

^{1}H NMR of FM-002

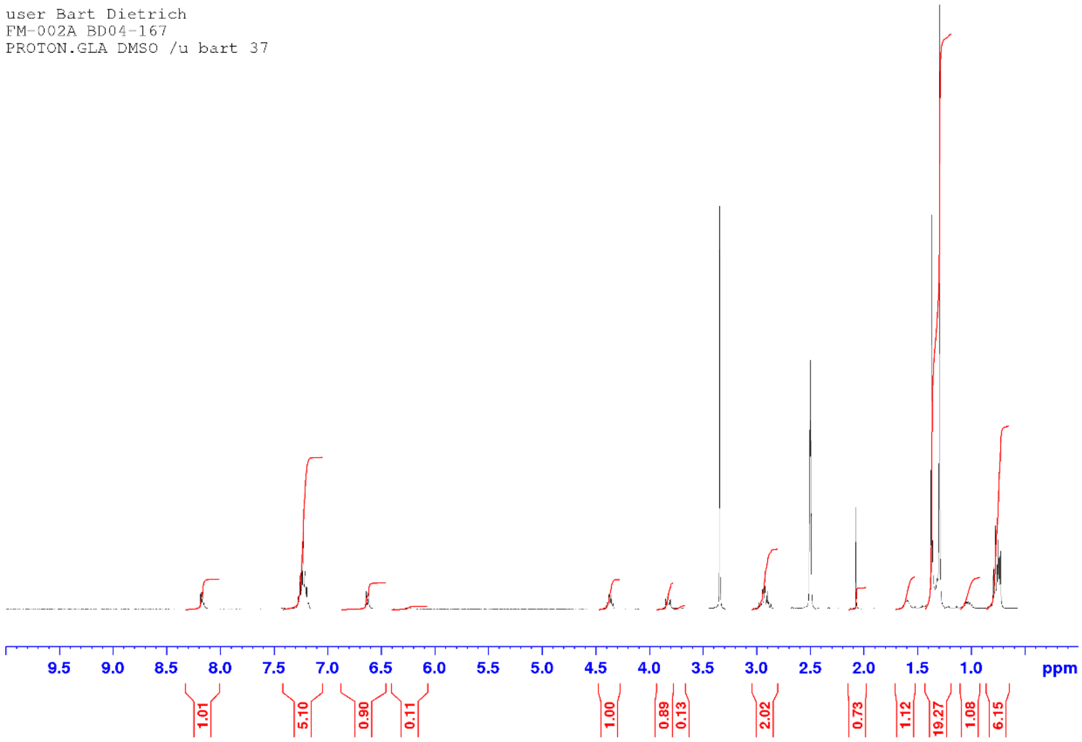

^{13}C NMR of FM-002

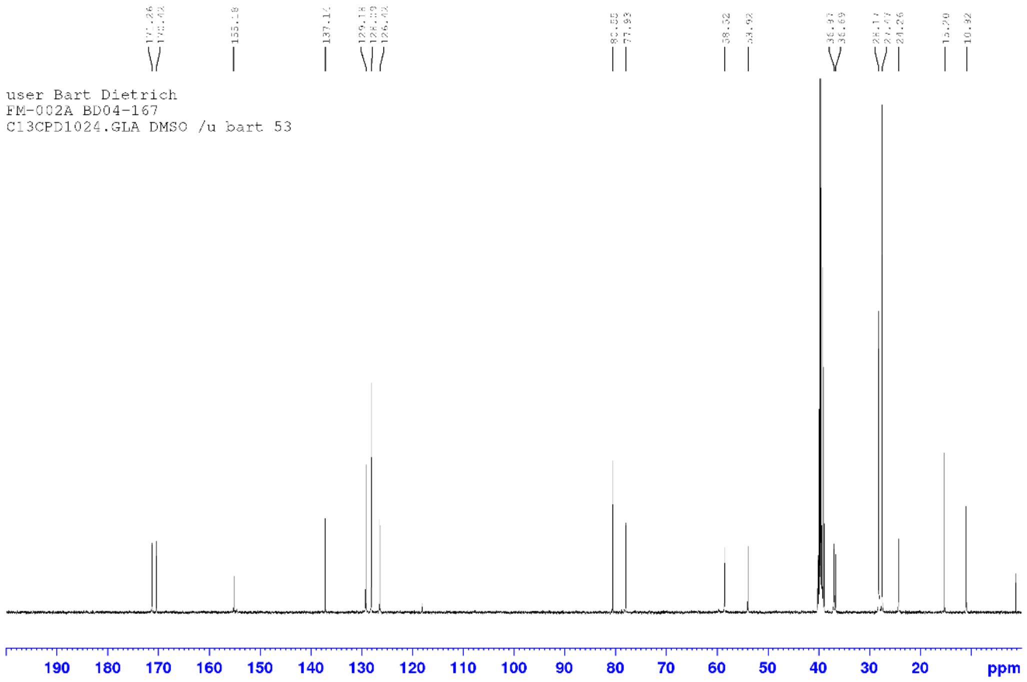

(2*S*)-2-[(2*S*,3*S*)-2-Amino-3-methylpentanamido]-3-phenylpropanoic acid hydrochloride (**8**)

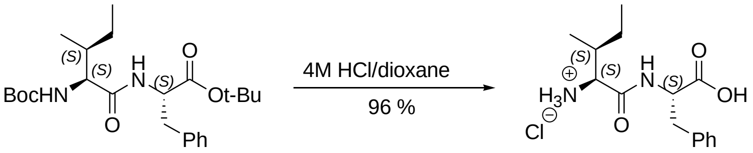

FM-002 **8**

FM-002 (6.40 g, 14.7 mmol) was dissolved in 4M HCl/dioxane (36 mL, *ca.* 10 eq) and stirred at ambient temperature for 3 days. After this time, the reaction mixture was poured into diethyl ether (*ca.* 150 mL) and evaporated under reduced pressure. To the sticky residue was added diethyl ether and the suspension was stirred vigorously overnight. It was then filtered and the solid in the filter washed with diethyl ether and dried under vacuum. The title compound was obtained as a white solid (4.42 g, 96%).

d_H (400 MHz, DMSO-d$_6$) 12.83 (1H, br s, COO<u>H</u>), 8.81 (1H, d, *J* 7.53, N<u>H</u>), 8.20 (3H, br s, N$\underline{H}_3^+$), 7.29-7.20 (5H, m, $\underline{H}_{Ar}$), 4.51-4.46 (1H, m, O=C-C<u>H</u>*), 3.68 (1H, d, *J* 5.19, O=C-C<u>H</u>*), 3.07 (1H, dd, *J* 14.05, 5.33, PhC$\underline{H}_2$), 2.97 (1H, dd, *J* 14.06, 8.57, PhC$\underline{H}_2$), 1.92-1.82 (1H, m, CH$_3$-C<u>H</u>*), 1.54-1.44 (1H, m, CH$_3$-C$\underline{H}_2$), 1.16-1.04 (1H, m, CH$_3$-C$\underline{H}_2$), 0.92 (3H, d, *J* 6.92, C$\underline{H}_3$-CH*), 0.84 (3H, t, *J* 7.36, C$\underline{H}_3$-CH$_2$). d_C (100 MHz, DMSO-d$_6$) 172.30 and 168.10 (<u>C</u>=O), 137.38, 129.23, 128.28, and 126.53 ($\underline{C}_{Ar}$), 56.29 and 53.93 (O=C-<u>C</u>H*), 36.43 (Ph<u>C</u>H$_2$), 36.34 (CH$_3$-<u>C</u>H*), 23.66 (CH$_3$-<u>C</u>H$_2$), 14.53 (<u>C</u>H$_3$-CH*), 11.31 (<u>C</u>H$_3$-CH$_2$). HRMS (ESI) m/z: [M+Na]$^+$ calcd for $C_{15}H_{22}N_2NaO_3$ 301.1523; found 301.1519.

^{1}H NMR of **8**

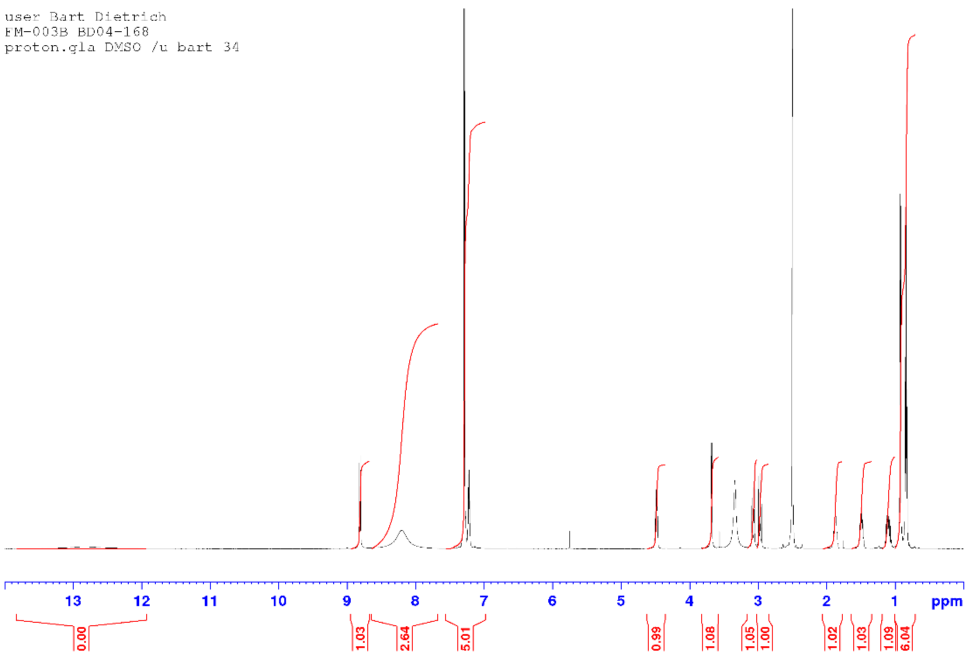

^{13}C NMR of **8**

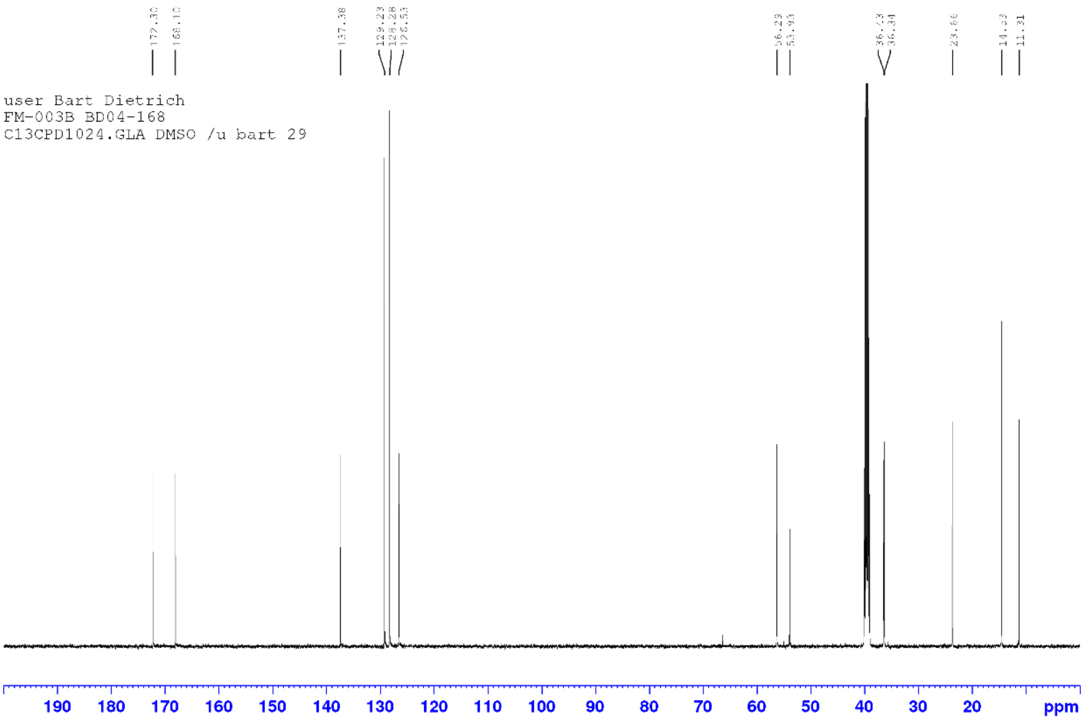

Preparation of solutions: Stock solution of **1** was prepared at a concentration of 10 mg/mL by dissolving in water in presence of 2 molar equivalents of NaOH (0.1 M). This solution was stirred overnight for complete dissolution of **1**. The final pH of the solution was then adjusted to pH 10. Aqueous solutions of **2**-**5** were prepared at a concentration of 5 mg/mL by stirring. Stock solutions of **6**-**8** were prepared in the concentration of 0.025 M in water. Stock solution of **9** was prepared at a concentration of 1 M in DMSO by heating at ~60 ^oC. Stock solution of **10** was prepared in water at pH 10 at the concentration of 5 mg/mL. Stock solution of GdL was prepared at a concentration of 100 mg/mL in H_2O and was used immediately after preparation. GdL is highly soluble in water and so did not require stirring. Stock solution of NaOH was prepared at a concentration of 0.1 M in H_2O.

Preparation of gels: A pH switch method was followed to prepare the gels involving **1**. 1 mL of the high pH solution of **1** (at pH 10) was transferred to a 7 mL Sterilin vial and further diluted by 0.9 mL of water. To this solution 0.1 mL of aqueous solution of GdL was added in one aliquot. The mixture was then left to stand overnight to allow gelation. Hence, in the system, the concentration of **1** was 5 mg/mL and the initial concentration of GdL was 5 mg/mL.

To prepare the hydrogels of **1** in presence of (**2**-**5**), a common procedure was followed. For these experiments, a mixture of 0.8 mL of Fmoc-salt (**2**-**5** as required), 0.1 mL of GdL and 0.1 mL of water was prepared and then immediately transferred to the vial containing 1 mL of **1** (at pH 10). The systems were then left undisturbed overnight for gelation. Therefore, in the respective gels, the concentration of **1** was 5 mg/mL, concentrations of (**2**-**5**) were 2 mg/mL and the initial concentration of GdL was 5 mg/mL.

Hydrogels of (**1**+**2**) were also prepared at different concentrations of **2**. For these experiments either a mixture of 0.4 mL solution of **2** and 0.5 mL of water or a mixture of 0.2 mL of solution of **2** and 0.7 mL of water was prepared first. To these mixtures, 0.1 mL of GdL was individually added and the solutions were then transferred to the vials containing 1 mL of solution of **1** (at pH 10). Therefore, in the respective gels, the concentration of **1** was 5 mg/mL, initial concentration of GdL was 5 mg/mL and the concentration of **2** was either 1 mg/mL or 0.5 mg/mL.

To prepare the gels in a syringe (12 mL), initially components were mixed at their respective volumes as mentioned earlier. It is to note that, solutions of **1** are free flowing both in absence and presence of **2** just after addition of GdL. 1 mL of these solutions are transferred into the syringe when the mixture of (**1**+**2**) became viscus after ~40 mins of addition of GdL.

Gelation tests for (**2**-**5**) were carried out in presence of equimolar amounts of NaOH at a concentration of 2 mg/mL. In all cases, the volume of the solvent was maintained at 2 mL. All samples were left undisturbed for ~18 hours before measurements were carried out.

To prepare the hydrogels of **1** in presence of 1 molar equivalent of the salts **6**-**8**, a mixture of 0.5 mL of the salt, 0.4 mL of H_2O and 0.1 mL of GdL was transferred to the vial containing 1 mL of **1**. To prepare the gels in presence of 0.5 molar equivalent of the salts, 0.25 mL of the aqueous solutions of the corresponding salts were used and the rest of the volume was adjusted by water. For the hydrogels of (**1**+**9**), a mixture of 0.9 mL of H_2O and 0.1 mL of GdL was added to the mixture of **1** (1 mL) and **9** (12.5 μL and 6.25 μL for 1 equiv. and 0.5 equiv. respectively).

The hydrogels of **10** were prepared by diluting 1 mL aqueous solution of **10** with a mixture of 0.9 mL of water and 0.1 mL of GdL. To prepare the hydrogels of (**10**+**2**), a mixture of 0.45 mL of **2** (concentration of stock solution of **2** is 7.8 mg/mL), 0.45 mL of H_2O and 0.1 mL of GdL was added to the 1 mL of aqueous solution of **10** at pH 10.

pH measurements: All pH measurements were carried out using a A FC200 pH probe from HANNA instruments fitted with a 6 mm x 10 mm conical tip. The stated accuracy of the pH measurements is ±0.1 pH units. For the gels involving compounds **1**-**5**, the reaction mixtures were prepared as described above at a 2 mL volume in a 7 mL Sterilin vial and the pH change was monitored with time. The temperature was maintained at 25 °C during the measurement by using a circulating water bath.

pK_a determination of **1** was carried out by recording the pH values after each addition of HCl (0.1 M) to the solution of **1** (concentration is 5 mg/mL) containing 2 molar equivalents of NaOH (0.1 M) in H_2O. During the titrations, the solution was stirred continuously to avoid gelation. The experimental temperature was 25 °C.

Rheological measurements: All rheological measurements were carried out on an Anton Paar Physica MCR 301 rheometer at 25 °C. Strain, frequency and time sweeps were performed using a vane and cup geometry. Strain sweeps were performed at 10 rad/s from 0.01 % to 1000 % strain. Frequency sweeps were carried out from 1 rad/s to 100 rad/s at 0.5 % strain. All gels were left ~16 hours before being measured. Time sweeps were performed at an angular frequency of 10 rad/s and with a strain of 0.5%. For all experiments, gels were prepared as mentioned earlier in 2 mL volume in 7 mL Sterilin vials.

Confocal microscopy: A Zeiss LSM710 confocal microscope (Zeiss, Gottingen, Germany) with an LD EC Epiplan NEUFLUAR 50X, 0.55 DIC (Carl Zeiss, White Plains, NY, USA) objective was used for imaging. All gel samples were prepared in presence of Nile blue (2 μL/mL of a 0.1 wt % solution in water). Samples were prepared in 7 mL Sterilin vials keeping the same volumes of the components as mentioned earlier. Then small amounts of the gels were deposited onto glass microscope slides. A cover slip was placed carefully on the gel before imaging. All the samples were excited at 633 nm using a He-Ne laser. Images were captured using Carl Zeiss ZEN 2011 v7.0.3.286 software.

UV-Vis measurements: Absorption spectra of **1**-**5** under different conditions were recorded on an Agilent Technologies Cary 60 UV-Vis spectrophotometer using a 0.01 mm path length quartz cuvette. All gel samples were prepared in Sterilin vials using the same methodology as described before and were left overnight. Then, small amounts of the gels were transferred to the cuvette for measurement.

To record the absorption spectra of the exuded water from the gels, 0.05 mL of the solutions were trasferred to a 0.1 mm path length quartz cuvette. Absorption spectra of 0.05 mL of aqueous solutions of **5** (2 mg/mL) and **2** (0.5 mg/mL) were also recorded using a similar procedure for quantitative analysis.

Fluorescence spectroscopy: Data were collected on an Agilent Technologies Cary Eclipse fluorescence spectrometer. Samples were prepared in PMMA cuvettes with a path length of 1 cm by following the same procedure as mentioned before. In all cases, the excitation wavelength was 350 nm. Both the excitation and emission slit widths were 5 nm.

FTIR spectroscopy: Data were recorded using an Agilent Cary 630 FTIR spectrometer (with ATR attachment). For gels, D_2O was used for the background correction. All gels were prepared in D_2O following the same methodology as described above. Then, small amounts of the gels were deposited on the ATR crystal before recording the spectra.

Supplementary Figures

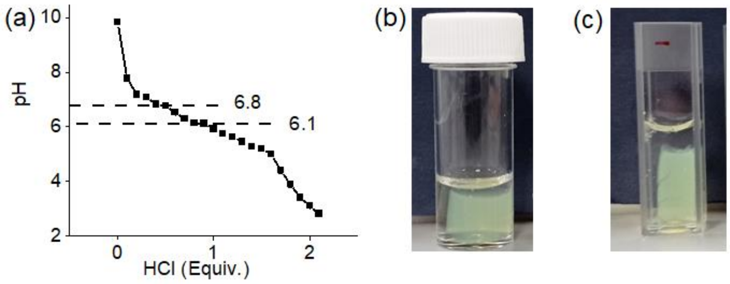

Figure S1. (a) Determination of apparent pK_a of **1** (5 mg/mL) water. The plateaus are taken to represent the apparent pK_a values, shown by the horizontal lines. (b, c) Photographs representing the shrinkage of the hydrogels of **1**. The gelation experiments were carried out in (b) vial and (c) cuvette. The gels shrunk in a 3D fashion and adopted the shape of the containers. In both cases, concentrations of **1** and GdL are 5 mg/mL. 2 mL of aqueous solution of **1** was used for gelation in both cases.

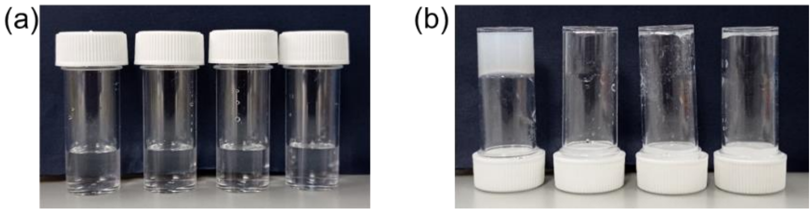

Figure S2. Photographs showing the phase changes of aqueous solutions of (a) **2**-**5** (from left to right in each photograph) in presence of equimolar amounts of NaOH (b). In all cases, concentrations of **2**-**5** are 2 mg/mL.

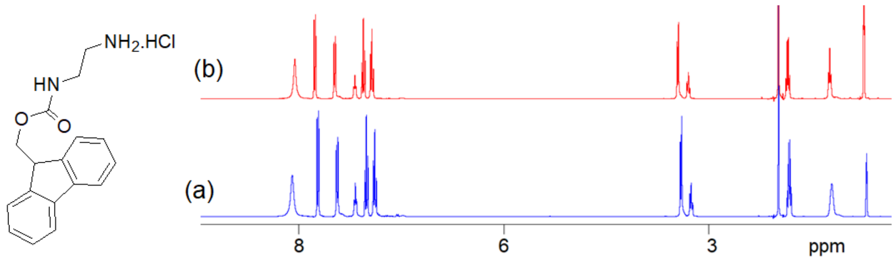

Figure S3. (a) Partial ^{1}H NMR spectrum of **2** (2 mg/mL) in d$_6$-DMSO. (b) Partial ^{1}H NMR spectrum of the freeze-dried sample of **2** (2 mg/mL) in d$_6$-DMSO. For (b), the sample was freeze dried after acidification of the hydrogel of **2** prepared at high pH. In (b), absence of any peak near 6.2 ppm corroborates no deprotection of the Fmoc-group.[3]

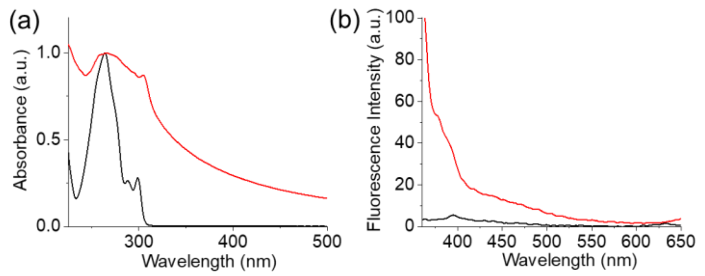

Figure S4. (a) Normalized absorption and (b) emission spectra (λ_{ex} is 350 nm) of **2** in absence (black) and presence (red) of equimolar amounts of NaOH in water. In all cases, concentration of **2** is 2 mg/mL.

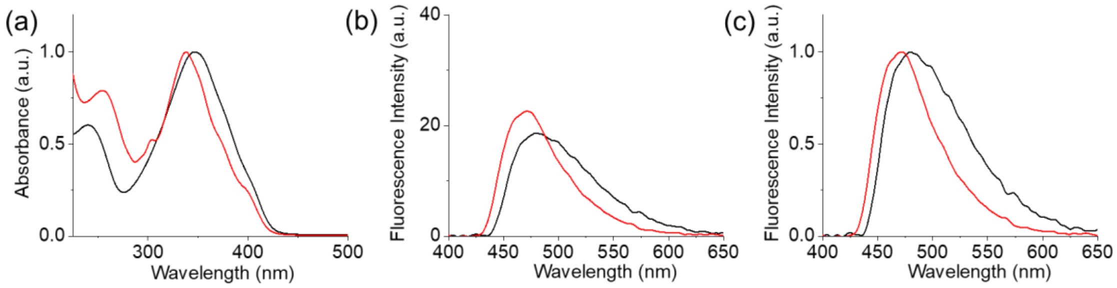

Figure S5. (a) Normalized absorption and (b) emission spectra (λ_{ex} is 350 nm) of **1** at high pH in absence (black) and presence (red) of **2** in water. Figure (c) is the normalized spectra of (b). In all cases, concentrations of **1** and **2** are 5 mg/mL and 2 mg/mL, respectively.

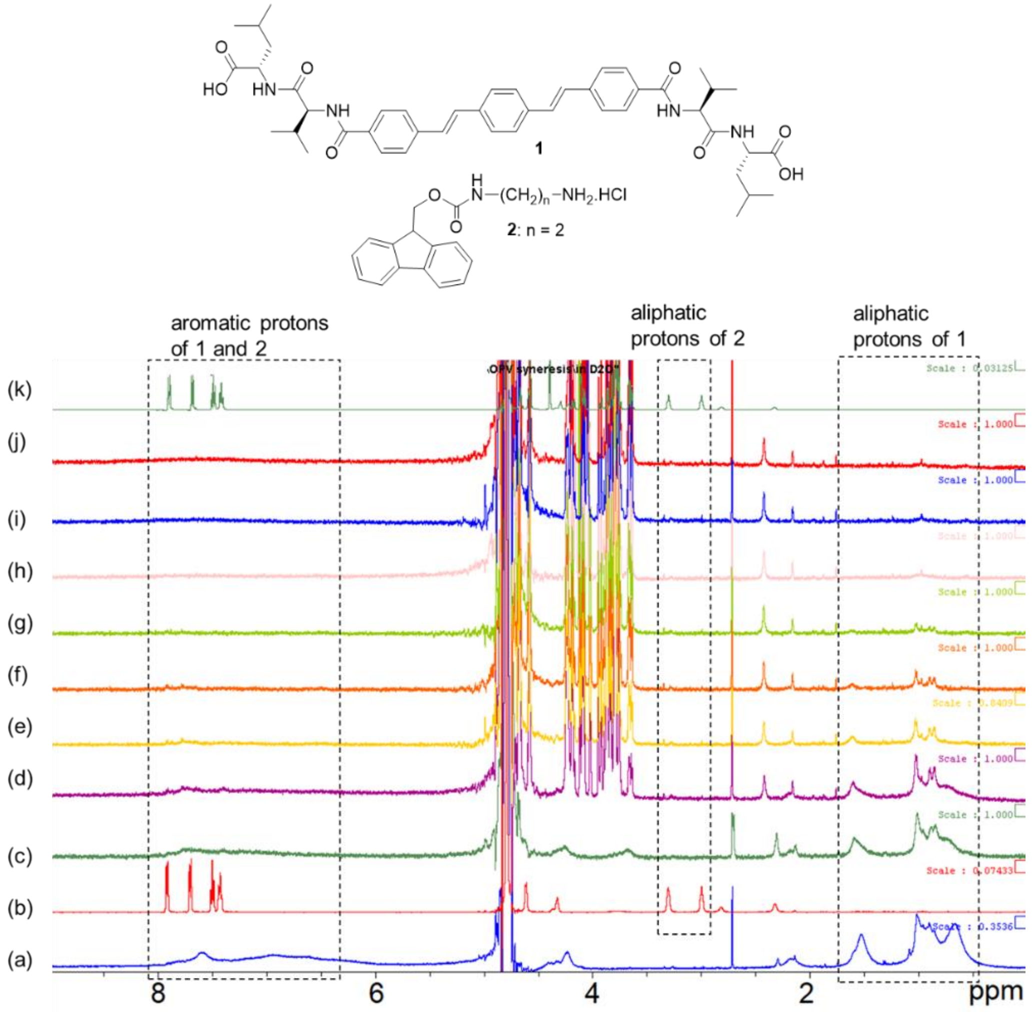

Figure S6. Partial ^{1}H NMR (500 MHz, D_2O, 25 ºC) spectra of (a) **1** at pH 10, (b) **2**, (c) (**1** + **2**) at high pH. (d-j) Changes in the proton NMR of the mixture of **1** and **2** upon addition of GdL at high pH: (d) immediate after addition of GdL, (e) 30 mins, (f) 1 h, (g) 2 h, (h) 5 h, (i) 10 h, (j) 16 h. (k) represents the proton NMR of **2** at low pH obtained involving hydrolysis of GdL. For (k), the spectrum was recorded after 18 h of addition of NaOH (1 molar equivalent) to the mixture of **2** and GdL. In all cases, concentrations of **1** and **2** are 5 mg/mL and 2 mg/mL, respectively. Initial concentration of GdL is 5 mg/mL. At high pH, the proton NMR of 1 is broad (a) due to the formation of micellar aggregates. However, it is identical to the reported spectrum of 1.[1]

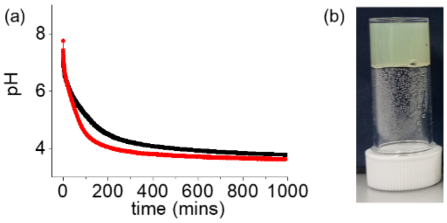

Figure S7. (a) Variation of pH with time for the solution of **1** (black data) and the mixture of (**1**+**2**) (red data) in presence of GdL. In both cases, concentrations of **1** and **2** are 5 mg/mL and 2 mg/mL, respectively. Initial concentration of GdL is 5 mg/mL. (b) Photograph of the hydrogel of (**1**+**2**) after 5 days. Concentrations of **1** and **2** are 5 mg/mL and 2 mg/mL, respectively. Initial concentration of GdL is 5 mg/mL.

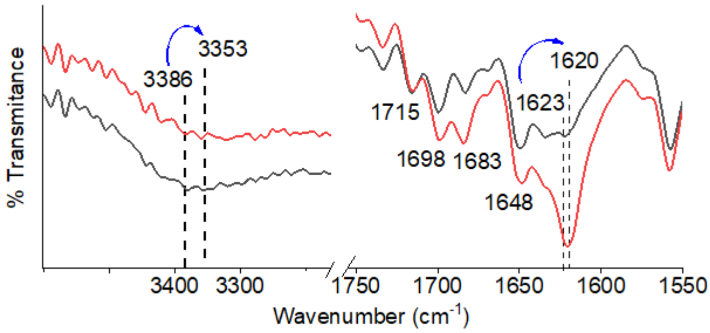

Figure S8. Partial FTIR spectra of the shrunken gel of **1** (black data) and the hydrogel of (**1**+**2**) (red data). Concentrations of **1** and **2** are 5 mg/mL and 2 mg/mL, respectively. Initial concentration of GdL is 5 mg/mL.

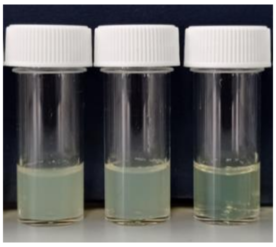

Figure S9. From left to right: photographs of solutions of **1** in presence of (**3**-**5**) in water at high pH. In all cases, concentration of **1** is 5 mg/mL, concentrations of (**3**-**5**) is 2 mg/mL.

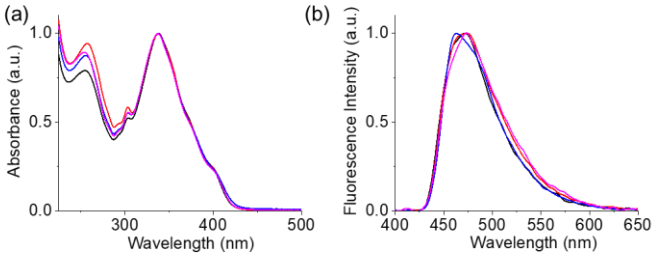

Figure S10. (a) Normalized absorption and (b) normalized emission spectra (λ_{ex} is 350 nm) for the mixtures of (**1**+**2**) (black), (**1**+**3**) (red), (**1**+**4**) (blue) and (**1**+**5**) (pink) at high pH in water. In all cases, concentration of **1** is 5 mg/mL, concentrations of (**2**-**5**) are 2 mg/mL.

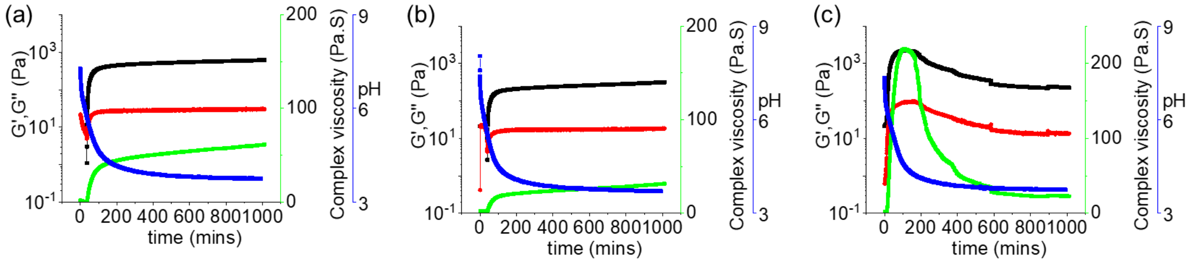

Figure S11. Variation of pH (blue), G′ (black), G″ (red) and complex viscosity (green) for the mixtures of (a) (**1**+**3**), (b) (**1**+**4**) and (c) (**1**+**5**) in presence of GdL in water. In all cases, initial concentration of **1** and GdL are 5 mg/mL, concentrations of **3**, **4** and **5** are 2 mg/mL.

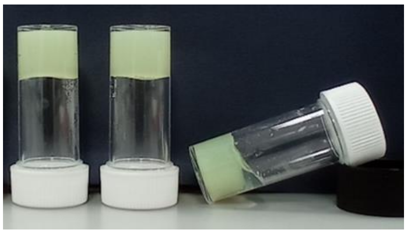

Figure S12. Photographs of the hydrogels obtained from the mixture of (from left to right) (**1**+**3**), (**1**+**4**) and (**1**+**5**) 18 hours after addition of GdL in water. In all cases, initial concentration of **1** and GdL are 5 mg/mL, concentrations of **3**, **4** and **5** are 2 mg/mL.

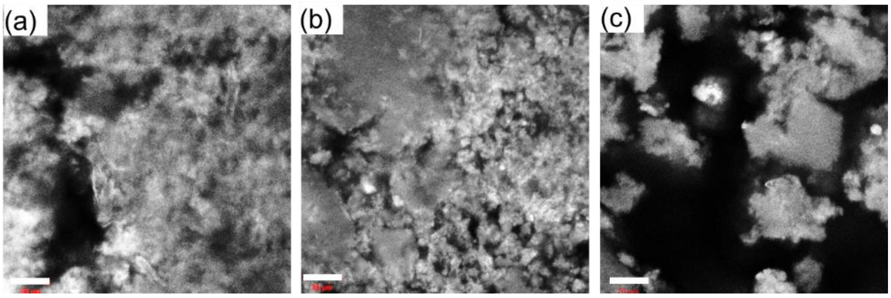

Figure S13. Confocal fluorescence microscopy images of the hydrogel of (a) (**1** + **3**), (b) (**1** + **4**), (c) (**1** + **5**) after 18 h. In all cases, the scale bar is 20 μm. In all cases, concentration of **1** and GdL are 5 mg/mL, concentrations of **3**-**5** are 2 mg/mL.

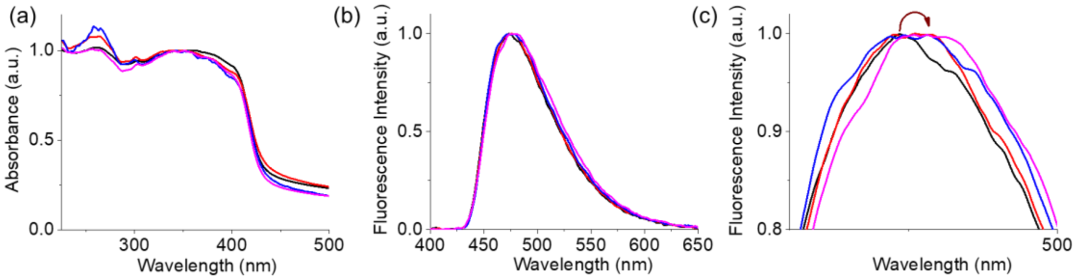

Figure S14. (a) Normalized absorption and (b) normalized emission spectra (λ_{ex} is 350 nm) for the hydrogels of (**1**+**2**) (black), (**1**+**3**) (red), (**1**+**4**) (blue) and (**1**+**5**) (pink) obtained involving the hydrolysis of GdL. Figure (c) shows expanded region of the graph (b). In all cases, concentration of **1** and GdL are 5 mg/mL, concentrations of **3**-**5** are 2 mg/mL.

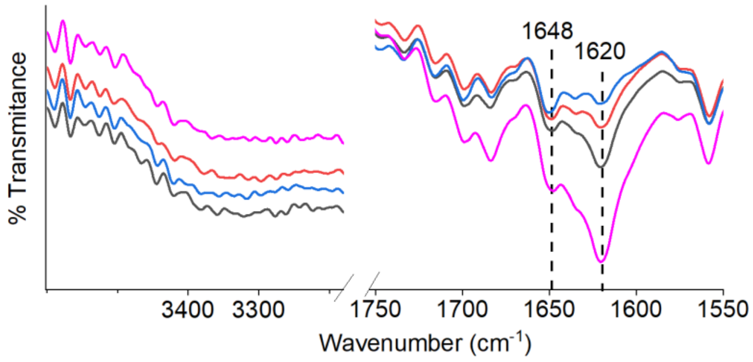

Figure S15. Partial FTIR spectra for the hydrogels of (**1**+**2**) (black), (**1**+**3**) (red), (**1**+**4**) (blue) and (**1**+**5**) (pink) obtained involving the hydrolysis of GdL. In all cases, concentration of **1** and GdL are 5 mg/mL, concentrations of **3**-**5** are 2 mg/mL.

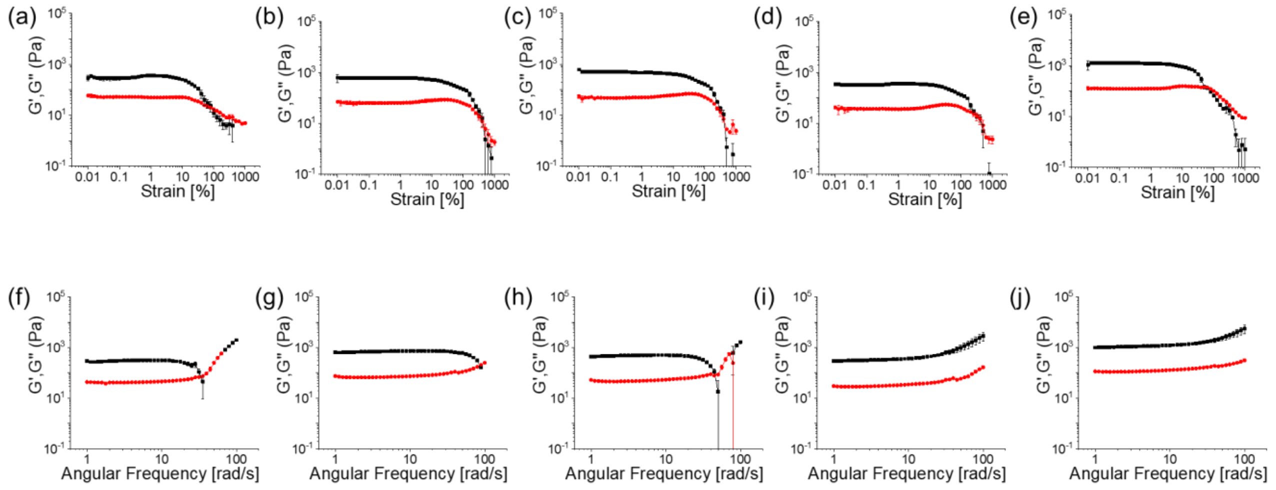

Figure S16. (a-e) Strain sweeps and (f-j) frequency sweeps of the hydrogels of **1** (a, f), (**1**+**2**) (b, g), (**1**+**3**) (c, h), (**1**+**4**) (d, i) and (**1**+**5**) (e, j) obtained involving the hydrolysis of GdL. In all cases, concentration of **1** and GdL are 5 mg/mL, concentrations of **3**-**5** are 2 mg/mL. The black symbols represent G′, the red symbols G″. Measurements were performed in triplicate (each measurement was done on a fresh new sample), and error bars were calculated from the standard deviation.

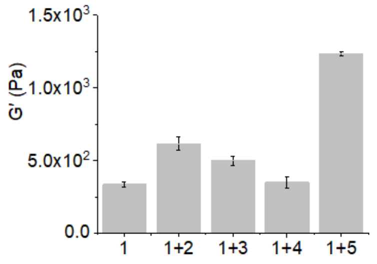

Figure S17. Bar graph representing the stiffness (G', calculated at 0.5% strain from strain sweeps) of the hydrogels obtained by using GdL. Concentrations of **1** and GdL are 5 mg/mL, concentrations of **2**-**5** are 2 mg/mL.

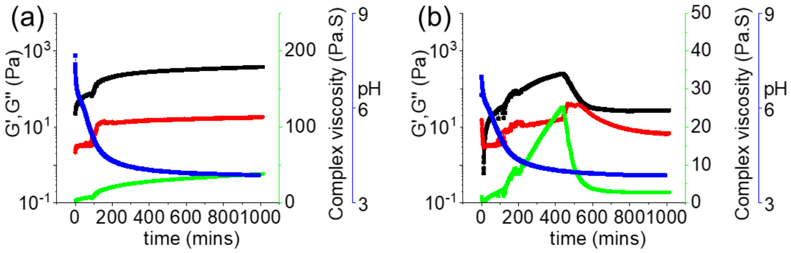

Figure S18. Variation of pH (blue), G′ (black), G″ (red) and complex viscosity (green) for the mixtures of (**1**+**2**) in presence of GdL in water. In all cases, initial concentration of **1** and GdL are 5 mg/mL. Concentration of **2** is 1 mg/mL (a) and 0.5 mg/mL (b).

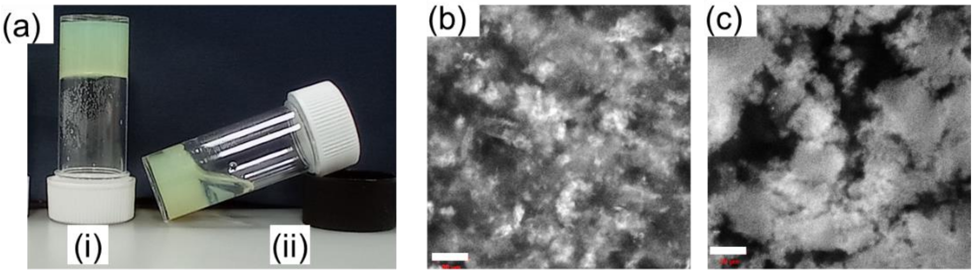

Figure S19. (a) Photographs of the hydrogels of (**1**+**2**) obtained in presence of GdL in water: (i) concentration of **2** is 1 mg/mL, (ii) 0.5 mg/mL. In both cases, concentration of **1** is 5 mg/mL, initial concentration of GdL is 5 mg/mL. (b, c) Confocal fluorescence microscopic images of the gels of (**1**+**2**) prepared using conditions (i) for (b), and (ii) for (c).

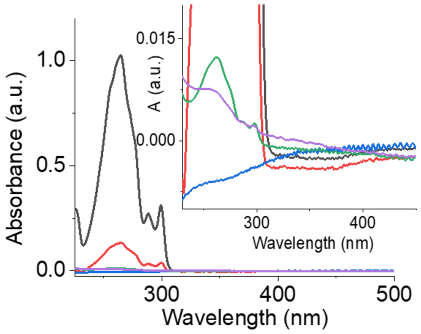

Figure S20. UV-vis spectra of the aqueous solutions of **5** (concentration 2 mg/mL) (black) and **2** (concentration 0.5 mg/mL) (red), and the solutions exuded from the hydrogels of **1** (blue), (**1**+**5**) (green) and (**1**+**2**) (purple). In the gels, concentrations of **1** and GdL are 5 mg/mL, concentrations of **2** and **5** are 0.5 mg/mL and 2 mg/mL, respectively.

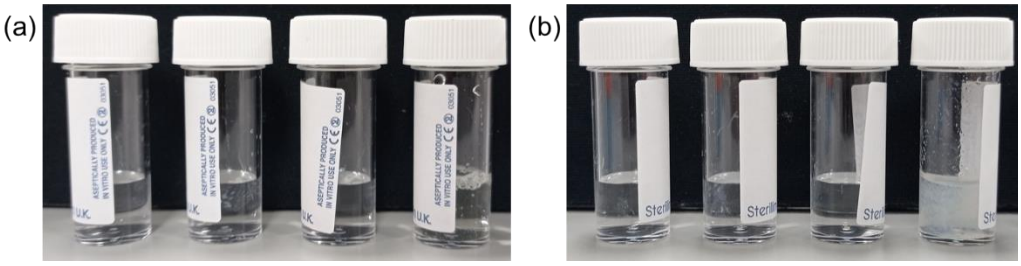

Figure S21. Photographs showing the phase changes of aqueous solutions of (a) **6**-**9** (from left to right in each photograph) in presence of equimolar amounts of NaOH (b). In all cases, concentrations of **6**-**9** are 6.25 x 10^{-3} M.

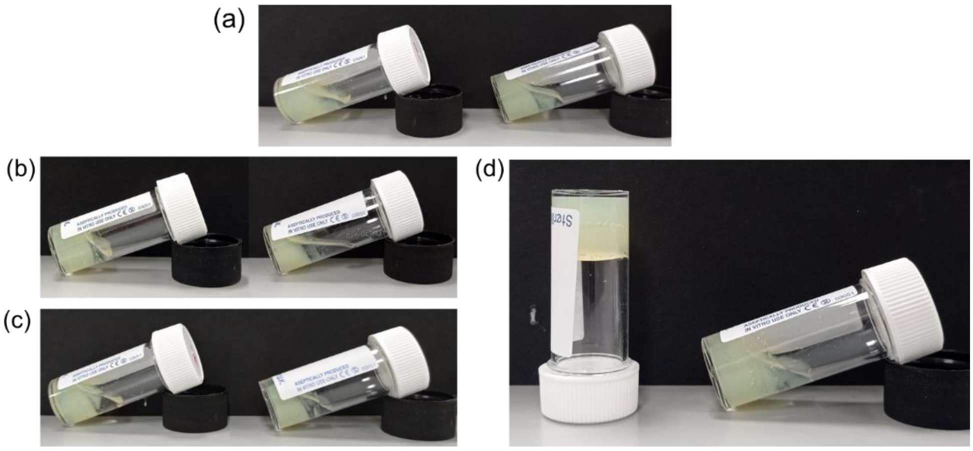

Figure S22. (a-d)Photographs of the hydrogels obtained from the mixture of (**1**+**6**), (**1**+**7**) and (**1**+**8**) and (**1+9**) 18 hours after addition of GdL. In each photograph, the left and right vials represent the systems **1** with 1 molar equiv. and 0.5 molar equiv. of the salts respectively. In all cases, initial concentration of **1** and GdL are 5 mg/mL.

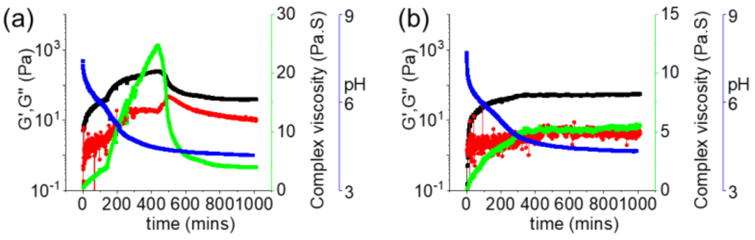

Figure S23. Variation of pH (blue), G′ (black), G″ (red) and complex viscosity (green) for the mixtures of (a) (**1**+**7**) and (b) **(1 + 9**) in presence of GdL in water. In all cases, initial concentration of **1** and GdL are 5 mg/mL. Concentrations of **7** and **9** are 1 molar equivalents with respect to **1**.

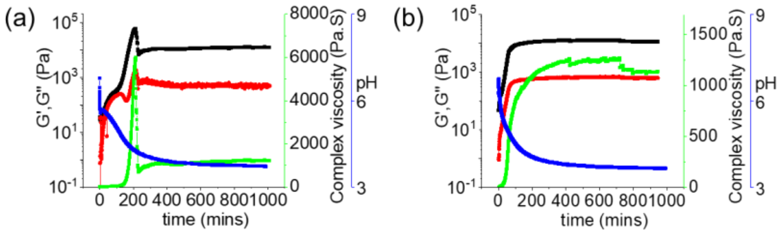

Figure S24. Variation of pH (blue), G′ (black), G″ (red) and complex viscosity (green) for (a) **10** and (b) the mixture of **10** (5 mg/mL) and **2** (0.5 molar equivalent) in presence of GdL in water. In both cases, initial concentration of GdL is 5 mg/mL.

References

[1] A. M. Castilla, M. Wallace, L. L. E. Mears, E. R. Draper, J. Doutch, S. Rogers, D. J. Adams, *Soft Matter* **2016**, *12*, 7848-7854.

[2] L. Chen, K. Morris, A. Laybourn, D. Elias, M. R. Hicks, A. Rodger, L. Serpell, D. J. Adams, *Langmuir* **2010**, *26*, 5232-5242.

[3] S. Panja, D. J. Adams, *Chem. Commun.* **2019**, *55*, 47-50.
